# EARLY-LIFE INFLUENCES ON LATER-LIFE HEALTH

**DOI:** 10.1093/geroni/igz038.218

**Published:** 2019-11-08

**Authors:** Sara M Moorman, Jacqui Smith

**Affiliations:** 1 Boston College, Chestnut Hill, Massachusetts, United States; 2 University of Michigan, Ann Arbor, Michigan, United States

## Abstract

This symposium identifies risk and protective factors in childhood and adolescence that continue to influence the physical and cognitive health of older adults. Dimensions of early life inequality include geographic location, educational opportunities, and total amount of adversity faced. Papers in the symposium use data from three major U.S. longitudinal studies: the Health and Retirement Study (HRS), the MacArthur Study of Midlife in the U.S. (MIDUS), and the Wisconsin Longitudinal Study (WLS). Each paper focuses on the mechanisms (i.e., mediators and moderators) linking well-being in childhood and adolescence to health in older adulthood. Greenfield, Akincigil, and Moorman find that net of IQ, college education boosts later life cognition, especially for men who had a low probability of college attendance. Kemp and Montez further find that state economic policy and adult health behavior explain later life disparities in health by educational attainment. Herd, Sicinski, and Asthana continue the theme of geographic place, finding that rural children who were raised on farms are at a cognitive disadvantage in later life. Both Ferraro and Sauerteig and Homan and Kong generate indices of total childhood misfortune. Ferraro and Sauerteig conclude that childhood misfortune affects obesity by way of adult health behavior, specifically, physical activity. Homan and Kong find that childhood misfortune affects subjective and functional health by way of the psychological mechanism of purpose in life. Together, these five papers begin to identify the ways in which experiences in childhood and adolescence have long-lasting consequences for a variety of health outcomes in later life.

